# Hungarian Fine-to-Coarse Aggregate, a Possible Constituent of Near-Vessel Structural Concrete of Nuclear Power Plants

**DOI:** 10.3390/ma16093520

**Published:** 2023-05-04

**Authors:** Katalin Gméling, Veronika Szilágyi, Ildikó Harsányi, László Szentmiklósi

**Affiliations:** Nuclear Analysis and Radiography Department, Centre for Energy Research, 1121 Budapest, Hungary; szilagyi.veronika@ek-cer.hu (V.S.); harsanyi.ildiko@wigner.hu (I.H.)

**Keywords:** sand, pebble, aggregate, shielding concrete, neutron and gamma-ray radiation, long-lived isotopes, nuclear analytical techniques

## Abstract

Significant gravel mines, representative of four regions of Hungary (northeast, central, northwest, and southwest) were systematically sampled to characterize their sand and pebbles as potential constituents of nuclear-grade concrete. The samples were analysed for their elemental compositions as a function of the mining locality and grain size, using two complementary neutron-based analytical techniques, prompt gamma activation analysis (PGAA) and neutron activation analysis (NAA). The combined analysis resulted in reliable mass fractions for over thirty elements that could be used to assess the radiation shielding and activation properties of the resulting concrete, essential in nuclear applications, by means of computer simulations. The studied aggregates are proven to be appropriate constituents for biological shielding at radiological centres, NPPs, and at nuclear research installations, even in mixed neutron/gamma radiation fields. The elemental compositions also revealed geochemical differences between the sedimentologically different regions.

## 1. Introduction

Nuclear Power Plants (NPP) produce energy by controlling the nuclear chain reaction of ^235^U. During the nuclear reaction, neutrons, gamma, alpha, and beta radiations are induced, besides the production of considerable heat. Neutrons cause structural damage to the exposed materials by secondary gamma and alpha radiation and via activation [1,2,3,4,5]. In tank-type reactors, a water moderator is used to slow down the neutrons and remove the heat. The tank is a steel vessel, which is not only stainless but also engineered to preserve its mechanical properties even amid high doses of neutron and gamma radiation, for several decades [1,2,3,4,5]. The vessel is surrounded by thick concrete biological shielding that is also exposed to neutrons that have leaked through the vessel [6,7]. Such concrete must resist severe gamma doses and elevated temperatures up to 70 °C, while neutrons activate this material [8,9]. Both the vessel and the near-vessel part of concrete must be designed to minimize radioactivation during their service lifetime and later during the decommissioning and waste management phase. The first aspect is important for minimizing the dose rate of personnel during maintenance tasks, while the latter has a strong impact on the post-operational costs.

The ALARA (as low as reasonably achievable) principle is a widespread design philosophy in nuclear science. In this case, the neutron-induced activation can be minimized by choosing appropriate raw materials that contain precursors of long-lived and highly-activating radioisotopes only in low amounts. Concrete is made of gravel and sand as aggregates, bonded with cement, water, and with other minor additives, and admixtures. Although the chemical properties of the mixture are determined by each constituent of concrete, in this paper we focus on the sand and gravel as aggregate components. Aggregates being the predominant component (approx. 60–75 m%; m% is mass percent), they affect most of the overall chemical composition, and they contain the most trace elements responsible for the activation character. Moreover, cement, water, and additives are quality-controlled industrial products, while mine products show considerable natural variability. Cement is the second major component of concrete, and thus the average cement composition is used as a basis for comparison. This calls for their qualification before using them in nuclear-grade concrete.

Regarding the neutron-induced activation of gravels and sands, the rare earth and microelement contents will be in focus, as they are responsible for the long-term radioactivity, as their daughter isotopes have long half-lives (e.g., ref. [10]). Alpha and beta particles have low mean free paths, so their contributions to the surface dose rate are limited; therefore, from a dosimetry point of view, gamma rays emitted during the radioactive decay are to be minimized. The selection of raw materials as additives and cement used in the shielding concrete depends on the type of radiation field. Light elements, especially hydrogen and boron, are appropriate against neutron radiation, while heavy elements are useful against gamma radiation. Concrete shields against neutrons contain low-density aggregates, such as volcanic tuff, limestone, perlite, blast furnace slag, and/or other industrial waste. Serpentinite can also be used as it has not only high hydrogen content but also relatively high Fe content, which is preferable against gamma radiation in a mixed neutron/gamma radiation field. The concrete designed for gamma shielding is called heavyweight if containing high-density aggregates with elements of a generally high atomic number (such as barite, magnetite, or industrial materials such as iron, lead shots, etc.).

After the reactor’s lifetime is over, its parts must be treated as radioactive wastes of different categories. The activity of radioactive waste drops after a few years of cooling as the short-lived nuclides decay. The rest of the radioactivity is more persistent, due to long-lived radionuclides, and therefore must be placed in special radioactive waste repositories, and their quantities, for economic and environmental-protection reasons, must be kept at the lowest possible. Research on radioactive waste management expanded in parallel with the test studies carried out in research reactors, where neutron activation techniques helped the detailed material analysis [11]. One aspect is to design certain parts of the shielding as separable pieces, to make the disassembling, maintenance, and transport easier. For example, the inner mantle of the concrete (the lightweight concrete) should be poured separately, as it will probably suffer the most severe radiation damage (displacement per atom, dpa) and can be physically destructed. In the case of a lifetime prolongation, the damage to parts of the biological shielding can be assessed piece by piece, separately. The thickness of the mantle should be determined by reliable simulations, to absorb all relevant radiation and protect the rest of the load-bearing concrete structure. Furthermore, ingredients of the near-vessel concrete parts should be chosen aiming for the lowest possible activation. Its activation tendency can be precomputed by considering the panoramic elemental composition of the relevant raw materials and their mixing ratios.

The concrete structures of the Hungarian nuclear reactors and their shielding will be made preferably using domestic raw materials. For this reason, we screened the compositions of sand and gravel materials from different sources in Hungary using analytical and petrographic [12] methods, and thereby we contribute to the development of a radiation-resistant, durable, and low-activation concrete.

## 2. Materials

To achieve our research objectives, we systematically sampled the Hungarian gravel and sand mines. The gravel and the sand are, from a geological point of view, debris sediments, which were transported and deposited by rivers; thus, the composition of sediments is determined by the site of origin, the distance of transport, and the depositional environment.

**Figure 1:**
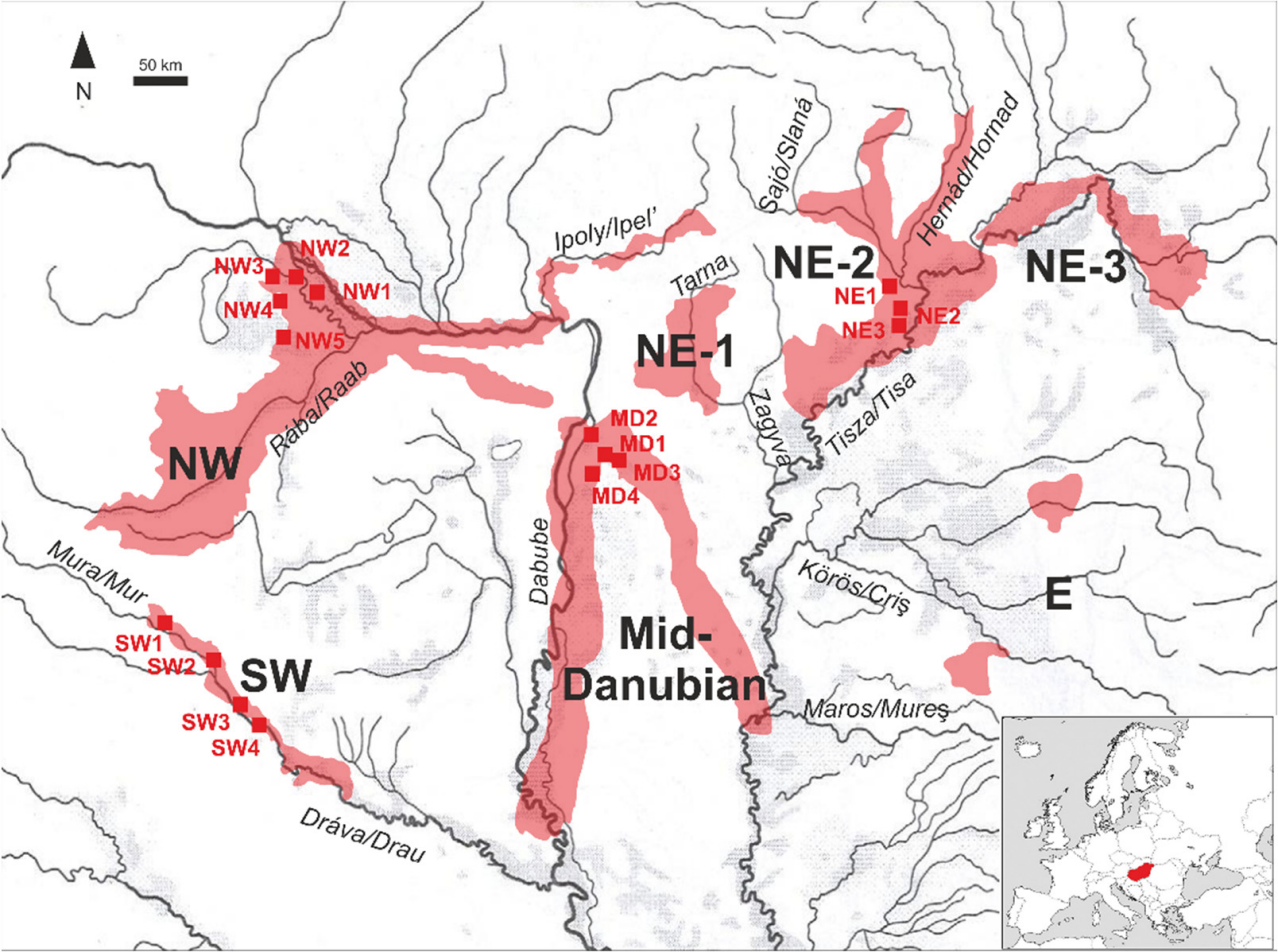
Major pebble mining regions in Hungary with the indication of samples studied in this paper: NW-Hungarian Region (upper section of river Danube and the valley of river Rába); Mid-Danubian Region (middle section of river Danube); SW-Hungarian Region (riverbed of river Dráva); NE-1 Region (alluvial cone of brook Tarna in the foreground of Mátra Mts.); NE-2 Region (alluvial cone of rivers Sajó-Hernád in the foreground of Bükk Mts.); NE-3 Region (alluvial Szatmár-Bereg plain of river Tisza); E Region (terraces of rivers Szamos, Kőrös, and Maros). The source of the hydrographic map of the Carpathian basin: http://pctrs.network.hu/clubpicture/1/3/_/karpat_medence_vakterkep_terkep_2_103364_22555.png, (accessed on 15 January 2023). The alluvial cone of rivers Sajó-Hernád in the southern foreground of the Bükk Mountains serves the largest pebble quarries of Hungary (NE-2 Region, mentioned later as NEHR, see Figure 1, e.g., Alsózsolca, Nyékládháza). The thickness of the clastic formation can even reach 100 m. The lithological composition is driven by the erosional area of rivers Sajó, Bódva, and Hernád which originate in the Low Tatras, the Slovak Ore Mountains, and the Gemer-Turňa Karst. Those are composed of granitoids and other crystalline formations of the Gemericum Unit [13], metaophiolites of the Meliaticum Unit [14,15], and carbonatic rocks of the Silicicum Unit [16,17,18].

The major pebble sources of Hungary were formed during the Quaternary (approx. last 2.6 million years) when, parallel with the continuous subsidence of the Carpathian Basin, the modern river network evolved. The marginal mountainous regions provided a large amount of coarse-grained clastic material for alluvial sedimentation deposited at the boundary of the mountainous and basinal territories in the form of alluvial cones. In addition, clastic sediments were also deposited on the alluvial terraces, which—due to the gradual incision of riverbeds—preserved the pebble formations on elevated areas. The result was the thick (tens-to-hundreds of meters) alluvial gravel formations, mainly located at the mountain verges, where the modern pebble quarries are situated (Figure 1). The quality and composition of the pebble formations were determined by the lithology of the erosional area. In the following, four major depositional regions, which we studied, are described in detail.

Alluvial sediments of the river Danube are quarried in Hungary at two distinct sections of the river. The NW-Hungarian Region (NWHR in Figure 1, e.g., Hegyeshalom, Mosonmagyaróvár) represents the middle-course type sedimentation of the river, with a high ratio of coarse-grained fractions and moderate (re)working of the grains, forming a huge alluvial cone having 100–250 m (max. 700 m) of Quaternary fluvial sandy-gravelly sediments [19,20]. In the Mid-Danubian region (MDR in Figure 1, e.g., Délegyháza, Bugyi), Pleistocene-Holocene terraces of the Danube are covered with fine-medium-coarse-grained sand and fine-grained gravels with well-rounded and medium spheric shapes [21,22,23]. The geology of the Danube’s catchment area is variable, due to the bulk transported by the Danube from the western mountainous regions and by the tributaries inside the basin representing local lithology [12]. The erosional area is dominated by metamorphics of the Bohemian Massif, the Alps, and the NW Carpathians. In addition, limestones of the Alps, the Leitha Mts., the NW Carpathians or Transdanubian Range, and younger volcanites of the Börzsöny-Visegrád Mts. are also present. Sediments of the River Dráva are quarried in the SW-Hungarian Region (SWHR see Figure 1, e.g., Gyékényes, Bélavár) in a relatively narrow area (30–40 km wide and 80 km long), which was formed by tectonic events approx. 20–2 million years ago, creating the Drava Trench [24,25]. The valley of Drava was filled up with fluviatile-alluvial sediments (some tens of m on the NW, 250 m on the SE) dominated by sandy–clayey formations but with one-third of coarse-grained clastic sediments. In addition, regional aeolian loess and glacial loam layers cover the surface of the eastern part [24,25]. The erosional area of the river is the central part of the Alps, which is characterised by crystalline rocks (granite, gneiss, mica schist, phyllite), shales, limestone, dolomite, and sandstone. Younger marine and river sediments (limestone and gravelly sandstone) are represented on the mountain margins.

From the four different regions (NWHR, SWHR, NEHR, and MDR), altogether sixteen mines were sampled. Four assorted and washed samples were collected from every selected mine. In total, 64 sediment samples were characterized for their elemental compositions. Four different fractions: sand and very fine gravels (0–4 mm), fine gravels (4–8 mm), medium gravels (8–16 mm), and coarse gravels (16–32 mm) were collected from each mine. Thus, twenty samples were selected from the five different mines from the North-West Hungarian Region (NWHR); four mines provided sixteen samples from the Mid-Danubian Region (MDR), and the other four mines had sixteen samples from the South-West Hungarian Region (SWHR), while three mines from North-East Hungarian Region (NEHR) made available twelve samples.

In addition, and for comparison, some binders (different types of cement), which is the other major component of concrete, from Vác and Királyegyháza cement factories, were analysed in the same way. The most-used cement type was Portland cement (CEM I), which is more than 95% clinker-calcinated (heated to 1450 °C) limestone with some amounts of clay. Three grades, differing in their compressive strength (32.5; 42.5; 52.5 MPa), each with initial strength N (normal) and R (rapid), were analysed.

## 3. Methods

Compositions of the samples were determined by the combination of two nuclear elemental composition analysis methods, neutron activation analysis (NAA) and prompt gamma activation analysis (PGAA). As they are based on the interaction of the neutrons with the matter, they probe exactly the set of isotopes that later determine the level of radioactivity. The element analysis measurements were performed at the Budapest Research Reactor (BRR) of the Centre for Energy Research [26,27]. The element identification relies on nuclear processes in the case of both techniques; therefore, their results are independent of the chemical composition and are of high volumetric representativity, reliability and metrological quality, and in this application, they outperform the more widespread X-ray fluorescence spectrometry. At our labs, we apply the *k*_0_-standardization method [28], which does not require a standard for the analysis.

NAA is for the quantitative determination of chemical elements down to ppm levels and is based on the partial conversion of stable nuclei in the sample to radioactive nuclei via known nuclear reactions. This is followed by the quantitation of the reaction products via their gamma radiations, in a separate step. The NAA is especially suited for trace element determination in the μg/g concentration range or below. The limit of detection is less than 0.01 μg for 30–50 elements, depending on the nuclear properties of the elements of interest, the measurement conditions, the neutron flux, density, and in some cases the matrix composition [28]. Up to 180 mg samples were weighed on a digital microbalance and sealed in high-purity quartz ampoules (Suprasil AN, Heraeus Quarzglas GmbH & Co.KG Division Base & Lamp Materials, Kleinostheim, Germany) for NAA. Samples were irradiated in a rotating, well-thermalized channel No. 17 of the BRR for 2–4 h, with a set of monitor foils: Zr, 0.1% Au in Al (IRMM 530 alloy), and Fe, to obtain the neutron flux parameters that are essential for the quantification using the triple bare monitor method [28]. The thermal neutron flux of the irradiation channel was measured at 1.86 × 10^13^ cm^−2^ s^−1^, with a relatively high thermal-to-epithermal ratio (*f* = 47). The gamma rays emitted from the samples were counted within iron low-level counting chambers, to reduce the room background. The HPGe detectors were connected to a dual-input ORTEC DSPEC 502 digital gamma spectrometer and controlled by the ORTEC Maestro 7 software. For spectrum evaluation, HyperLab 2013.1 software was used [29]. For the identification of radioactive isotopes and composition calculations, the KayZero for Windows 3.06 program [30] was applied.

During PGAA measurement, the sample is irradiated in a guided external neutron beam, when nuclei of the sample capture neutrons and characteristic prompt-gamma radiation is emitted. The irradiation and the measurement here are simultaneous. With PGAA, the major elemental composition (Si, Ti, Al, Fe, Mn, Mg, Ca, Na, K, H) of the samples and some special trace elements (B, Cl, Sc, V, Nd, Sm, Gd), which are hard to measure with other techniques, can be determined. PGAA is a panorama analysis technique and is a unique way to measure some light elements relevant to the nuclear industry, especially boron or hydrogen. It gives bulk composition and does not require sample preparation. The PGAA facility has been described in detail in an earlier publication [27]. The prompt spectra are evaluated with Hypermet-PC [31,32,33] and PeakFit gamma-spectroscopy software version 1.0 [34,35]. (Typical NAA and PGAA spectra are presented in the Supplementary Material) The element identification and quantification are carried out with the ProSpeRo program [36], utilizing our in-house developed and validated prompt-gamma analysis library [37]. Approximately 2–3 g powdered samples were weighed and heat-sealed into 25 mm thick Polytetrafluoroethylene (PTFE) bags of 2 × 3 cm. The neutron flux at the sample position of the PGAA station was about 7.7 × 10^7^ cm^−2^ s^−1^. The cross-section of the impinging neutron beam was adjusted to between 10 × 10 mm^2^ and 20 × 20 mm^2^, to optimize the count rate of the gamma detector, to keep the deadtime low enough (<5–7%) to be able to properly evaluate the gamma-spectra by peak fitting. The typical count rate was around 1000–1200 cps. The samples were analysed in an air atmosphere, the beam background of which was adequately corrected for [38]. The acquisition times were between 2500 and 58,500 s.

Although we determine the most relevant gamma-emitting isotopes with the above-mentioned techniques, there are other types of induced radiation. Alpha radiation is significant and it has high energy transfer, but as its mean free path is very short, ~10 µm, it causes structural damage only at the site of interaction in the material. This can form small particles, which can contaminate the air and be inhaled when getting into the gas phase. ^14^C, which is responsible for the largest part of the overall activation, is produced from ^14^N, ^17^O, and ^13^C, and hence it cannot be avoided. Its measurement is cumbersome and is not possible with neutron activation methods, due to the lack of gamma radiation. The case is similar to pure beta emitters, but has to be taken into account in the isotope inventory; hence carbon, chlorine, and phosphorous are also unavoidably present in concrete. The effect of beta radiation is structural too, as it is absorbed in the surrounding materials, up to a few hundred µm to a few mm, as its mean free path is longer than those of alphas. Although they cannot be directly measured with NAA, a good estimation can be given by simulating the complete activation process based on the equations; for example, in our case, by the FISPACT code [39].

Simulations in general are useful tools to validate our theoretical background knowledge and model theories by comparing them with experimental data so that later extrapolations, effect combinations, estimations, and predictions can be made on the systems. In our case, the complete set of activation processes can be described by the deterministic FISPACT code [39], based on the measured elemental compositions of gravel, model neutron flux, and corresponding nuclear data [40], by solving the Bateman equations of (n,γ), (n,α) and (n,β) and considering the subsequent decay processes [41]. The output results are the full isotope inventory with activity data, heat production, and DPA effects for each isotope, and the decay profile of each isotope can be visualised and compared for each component of the samples in time. The advantages of this code are described elsewhere [39]; for now, the wide range of applicable flux types and the different compilations of nuclear data are important. In our case, alpha and beta radiation can only be estimated by simulations, as our detector system is only sensitive to gamma rays. To assess the contributions of gravel and sand constituents to the activation of the near-vessel concrete, numerical simulation by FISPACT were carried out. The uncertainty of the simulation can be greater than the uncertainty of the measurement, as it also carries the uncertainty of the measured values and the uncertainty of the flux calculation. Simulation results, even if they come with higher uncertainties, can be compared to each other, assisting the identification of the most adequate raw materials. The simulation method has been validated earlier, with irradiation experiments [41]. This time, the experimentally obtained elemental compositions were combined with a distribution of neutrons modelling the probable neutron flux reaching the concrete wall outside of the steel vessel where the fast neutrons are already mostly thermalized. The flux was chosen to be higher than that of the power reactors, 2.6 × 10^13^ cm^−2^ s^−1^, representing the circumstances of accelerated aging studies typical of our research reactor. A continuous irradiation period of 10 years in our research reactor is equivalent to the real irradiation aging of concrete materials in a real power plant over 50 years. The output was set to print the activities of 1, 2, 5, 10, 25, 50, and 100 years following the 10-year irradiation, normalized to 1 g of sample mass, representing the layer of the real shielding concrete that is towards the source of the radiation. The real concrete absorbs most of the radiation dose in a few tens-of-centimetres depth; the outer part of the shielding block will not suffer so much structural damage and also will be less activated. In the thicker outer part, almost no neutron-generated activation is experienced, meeting the definition of biological shielding. 

## 4. Results

### 4.1. Elemental Composition Analysis

Altogether, 64 samples were analysed with NAA and PGAA methods from the four pebble mining regions of Hungary. PGAA provided concentrations of all the major element oxides (SiO_2_, TiO_2_, Al_2_O_3_, total Fe expressed as Fe_2_O_3_, MnO, MgO, CaO, Na_2_O, K_2_O, and H_2_O), while NAA gave the most trace elements to be compared. Thereby, the variation of the elemental composition among the four regions and among the different grain-size fractions in every region can be examined. As the chemistry of the sedimentary rocks can be quite variable, the element distributions are generally normalized to geological reference materials, to gain a more comparable picture. The most accepted reference is the average composition of the upper continental crust (UCC) [42,43]. The normalized data can present the enrichment or depletion of the given oxide compared to the average clastic material of the upper continental crust.

#### 4.1.1. The Major Oxide Distribution of the Samples in the Four Mining Regions

We found that the major oxide contents of the examined sands and gravels are depleted, relative to the UCC, except for their SiO_2_ contents, which are enriched. The pattern of the major element oxide concentration in the four regions shows overall similarity, although there are slight differences according to the origin and the sedimentary environment. Here we show the four regions one by one, and finally, compare them to each other.

**Figure 2:**
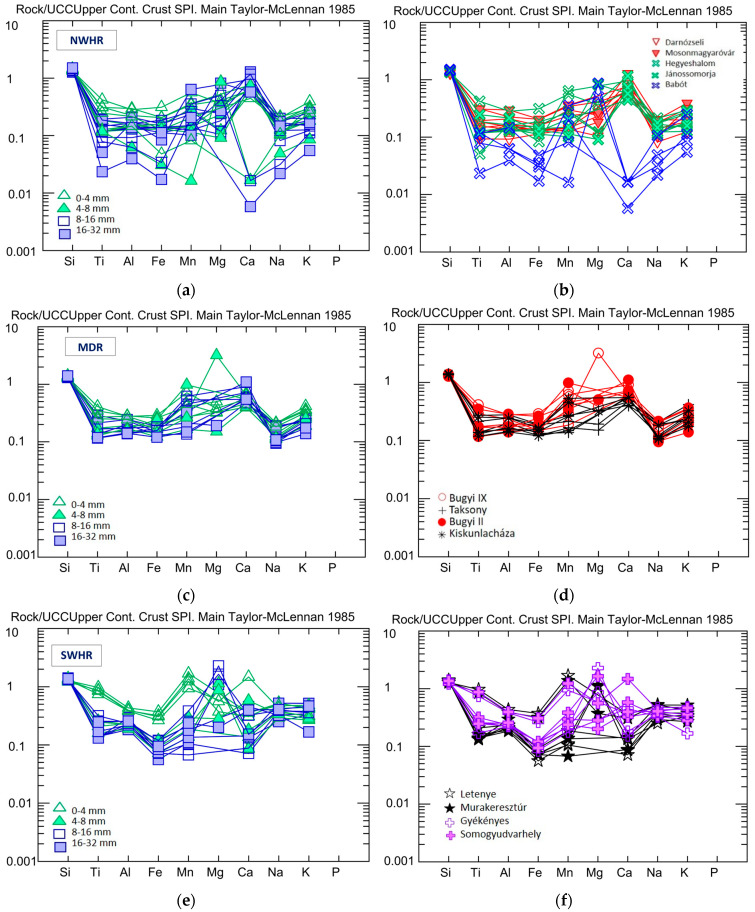

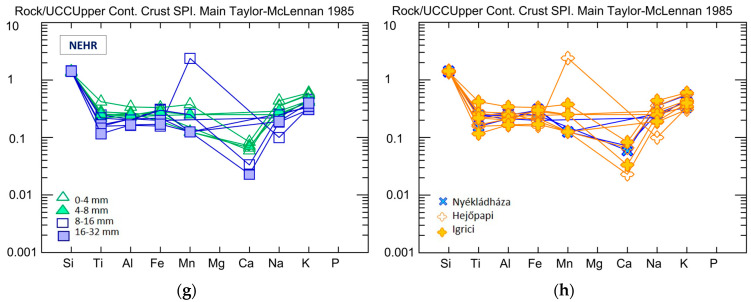
Major element-oxide distribution relative to the upper continental crust (UCC) average [42], in the NWHR, MDR, SHWR, and NEHR respectively, by grain size (**a**,**c**,**e**,**g**); and by the different mines (**b**,**d**,**f**,**h**).

At the ***NWHR***, all the major oxides show depletion, except for SiO_2_ (Figure 2a,b). The SiO_2_ content is above 84 m% and it can even reach 99 m%. The other major elements show wide variation in the examined 20 samples. Considering localities (Figure 2b), the samples from the Babót mine are spectacularly separated by their relatively low major oxide content (only enriched in SiO_2_). Babót is highly depleted in Ca and enriched in Mg, compared to other mines.

At the ***MDR***, the SiO_2_ content is above 83 m% and up to 93 m%. Except for the highly variable MnO and MgO, other major elements show much less variability in the analysed 16 samples (Figure 2c,d) than those in the NWHR (Figure 2a,b). Very slight enrichment of the Ti, Al, Fe, and K in the 0–4 mm fraction compared to coarser fractions is also visible here. There is not much correlation between the locality and elemental distribution (Figure 2d). All the major oxides show depletion, except for SiO_2_ (and MgO, in one case).

The ***SWHR*** samples have a SiO_2_ content between 80 m% and 93 m%. The major element concentrations show wide variation (Figure 2e,f), just like those in the NWHR. Significant enrichment of Ti, Al, Fe, and Mn oxide in the 0–4 mm fraction compared to coarser fractions is visible (Figure 2e). MgO and CaO show wide variation in the samples. There is no correlation between the concentrations and the localities (Figure 2f). All the major oxides show depletion compared to the UCC, except for SiO_2_ (in all samples), MnO (in 0–4 mm fractions), and MgO (in 8–16 mm fractions).

At the NEHR, the SiO_2_ content is between 89 m% and 95 m%. The other major elements show a lower degree of variability and depletion (Figure 2g,h), just like those in the MDR. There are no MgO data for these samples as the concentration was below the detection limit; in addition, their CaO contents are also very low. Slight enrichment of all major oxides is visible in the 0–4 mm fraction compared to other grain-size fractions. However, there is no correlation between the localities and element distributions Figure 2h.

#### 4.1.2. Trace-Element Concentration Variability in the Four Mining Regions

The trace element concentrations of the samples are found to be generally variable; their distribution is also examined relative to the average UCC. The normalized distribution diagrams line up the elements with increasing geochemical compatibility, with the most incompatible elements (i.e., tending to be concentrated in the melt phase during crystallization of the rock [44]) at the beginning of the row. Trace element distributions between the four different regions show a wide variety, but most of them are depleted compared to the average UCC, i.e., giving values lower than one in the graphs below.

**Figure 3:**
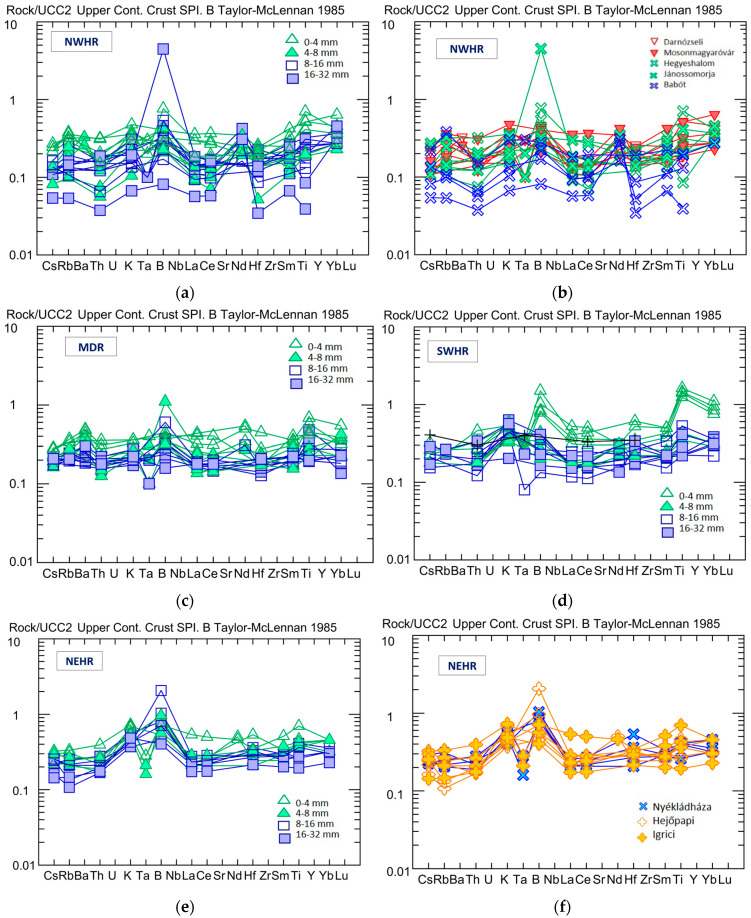
The trace-element distribution, relative to the upper continental crust (UCC) average [42], in the NWHR, MDR, SHWR, and NEHR, by grain size (**a**,**c**–**e**) and by the different mines in the case of the NWHR (**b**) and the NEHR (**f**).

All trace elements show depletion in the ***NWHR*** samples compared to the UCC average and are widely varied. The 0–4 mm and the 4–8 mm grain-size fractions are slightly enriched in trace elements (Figure 3a,b) compared to the coarser fractions. Considering the localities (Figure 3b), it is conspicuous that the trace elements in the samples from different mines differ by a factor of four.

There is a greater separation in the trace element concentrations according to the grain size within the ***MDR*** samples (Figure 3c). The 0–4 mm fractions are more enriched in these elements than all other grain sizes. The slight enrichment of B is interesting in some of the samples, but this is still low enough not to affect the neutronic behaviour of the resulting concrete.

At the ***SWHR***, the trace elements more compatible than Ta on the diagram (B, La, Ce, Hf, Nd, Sm, Ti, and Yb) are enriched in the 0–4 mm fraction (Figure 3d). B, Ti, and Yb in the 0–4 mm fractions were enriched, even compared to the concentration in the average UCC.

There are not as wide variations in the trace element distributions in the ***NEHR*** (Figure 3e,f) as in the other regions, and no close correlation with the grain size (Figure 3e), except in the quarry of Igrici, where the very finest fractions are relatively enriched in trace elements compared to other grain sizes (Figure 3f).

#### 4.1.3. Distribution of Trace Elements with Long Half-Lives Relevant to Waste Management

There are some elements (Ce, Hf, Fe, Sb, Tb, Sc, Ta, Cs, Co, and Eu) typically detectable in the ingredients of concrete which may form radioisotopes with longer than a month half-life after neutron capture [10]. Thus, their mass fractions are relevant to the case of residual activity investigations. Even without modelling the activity properties, the half-life gives a good indication of the isotopes that should be avoided in shielding concrete. There are three of the above-listed elements which have isotopes with remarkably long half-lives; these are Cs, Co, and Eu. The radionuclides formed from these elements show significant activity even years after the concrete was exposed to neutron radiation. All these trace elements are enriched in so-called heavy minerals (e.g., ilmenite, magnetite, amphibole, rutile, pyroxene, zircon), which are accessory constituents (<5%) of the sand and gravel, and which have a density of over 2.9 g/cm^3^. Heavy minerals mainly occur in sediments with lower quartz content (poor in quartzite and rich in other metamorphic, or igneous and siliciclastic rocks). We examined the concentration of these long-lived isotopes in all the studied sands and gravels of the four regions and in some batches of cement products. Portland cement (PC) is the most widely used binder in the concrete industry, and we gave the average element concentrations from the examined PC samples and presented them on the diagrams, for comparison with sand and gravel, as the other major constituents of concrete. The elemental compositions of the CEM I Portland cement samples reflect the lowest mean radiological-hazard indices, as also reported in the literature [45].

We note here that all samples were crushed with a jaw crusher, ground with a disk vibrating mill for homogeneity, and dried before elemental analysis. The jaw crusher and grinder had only a tungsten carbide (WC) tool, which can cause contamination of the samples [46,47,48]. Thus, we also used a different sample preparation technique on a series of samples, to see the level of contamination. We crushed four different-grain-size samples by hand and powdered them with an agate ball mill before the measurements, to see the possible contamination effect of the WC tool. We found that the samples that had been crushed with the WC tool contained around 0.5 m% W, and the amounts of Co and Ta also increased in those samples, but there were no changes in the concentrations of the other detectable elements. The use of a tungsten carbide tool can still influence the concentration of C in the samples, but the C concentration is below the detection limit of PGAA, and as we only applied long-irradiation NAA we could not detect the C with this method. Co, Ta, and W concentrations show a systematic increase with the level of contamination; thus, the Co and Ta content of those samples which were crushed with a WC-jaw crusher had to be corrected with a proportional correction factor.

**Figure 4:**
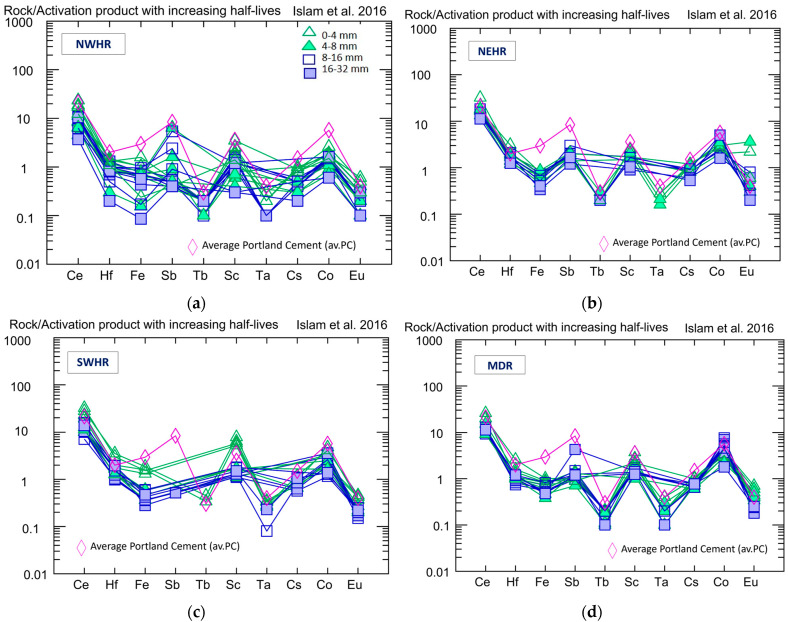
Trace element content in µg/g on a logarithmic scale, and its distribution in the different grain-size samples for the four examined regions ((**a**) **NWHR**; (**b**) **NEHR**; (**c**) **SWHR**; (**d**) **MDR**). Elements on the X-axis from left to right have increasing half-lives [10].

At the ***NWHR,*** the concentration of the long-half-life elements shows a wider variety than in other regions (Figure 4a), but all are below the average for the Portland cement. The concentrations of the long-lived tracers within the ***NEHR*** do not vary much (Figure 4b), and most of them are below the average for Portland cement. 

The ***SWHR*** is where these elements show enrichment in the 0–4 mm samples (Figure 4c) compared to other grain-size fractions, but in most of the cases, these are below the average for the Portland cement, except for Ce, Hf, and Sc, where they are slightly above. ***MDR*** shows little variation in the concentration of the listed long-lived isotopes (Figure 4d), and all data are below the average for the Portland cement, except for the Eu in the 0–4 mm grain size.

Cs, Co, and Eu have half-lives that are years long (^134^Cs T_1/2_ = 2.07 years; ^60^Co T_1/2_ = 5.27 years; ^154^Eu T_1/2_ = 8.5 years; and ^152^Eu T_1/2_ = 13.5 years), and these are the elements forming the focus of this study. The Cs and Eu contents are quite low, below 1 µg/g in every region and every grain size, except for a few cases in the NEHR. The Co mass fractions on average are below 5 µg/g in every region and every grain size. In some samples of the NEHR and MDR, the Co mass fraction can exceed 5 µg/g, but is still below 10 µg/g. Higher Co contents (>50 µg/g) were measured only in samples crushed and powdered with a WC tool, due to its contamination effect, but after applying the correction factor it is visible that the sand and gravel samples do not contain more than 10 µg/g Co.

### 4.2. Simulation Results

**Figure 5:**
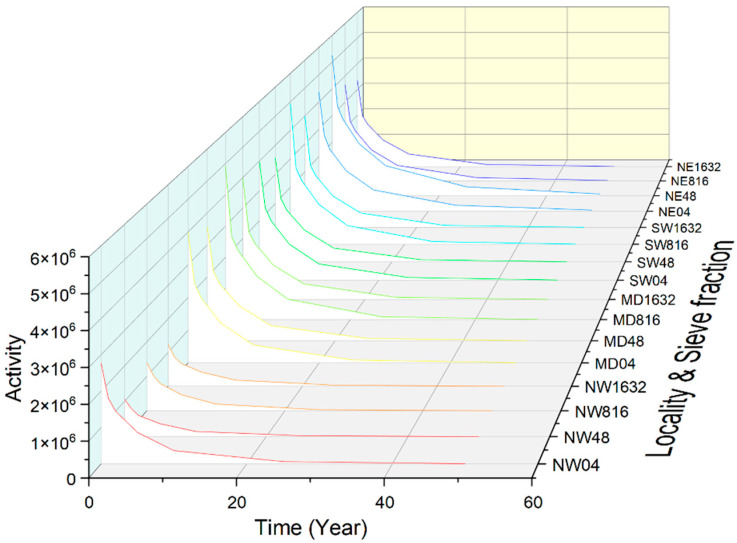
The decay of major long-lived isotopes of Co, Cs, and Eu in 60 years can be followed on the graphs by mining regions and grain-size fractions. Note that the initial differences up to a factor of five diminish only after about 30 years.

Based on the micro- and macro-element compositions, the decay or cooling of aged gravel and sand samples, as potential constituents of concrete, can be modelled. The decay over a 60-year timespan can be followed on the graphs by regions, quarries, and sieve fractions (Figure 5). To illustrate the long-term decay trends, we present the cumulative activities of the four most relevant long-lived isotopes that we can measure: ^60^Co, ^134^Cs, and ^152,154^Eu. These active nuclides are produced from elements Fe, Co, Cs, Sm, and Eu.

As is visible in Figure 5, there are slight differences among the regions and even smaller deviations between the fractions. The NWHR samples represent the lowest, while the other three regions display similarly higher activity. A slight but clear difference can be observed among the 0–4 mm and the coarser fractions of the NWHR and the SWHR, i.e., the finest sand–gravel samples show slightly higher activity. On the contrary, the coarser (8–16 and partly 16–32 mm) fractions become more radioactive in the MDR, compared to the finer samples.

## 5. Discussion

The sediment originating mainly from the Alps in the NWHR and SWHR shows the greatest variety in its major- and trace-element concentrations. This variety is due to the varying host rocks, the different transportation routes, and the sedimentation environment. At the NWHR, the element distribution is more influenced by the locality, while in the SWHR it is influenced by the grain size of the gravels. At the MDR and NEHR, the major-element concentrations of the samples are not very variable and do not show separation by grain size or the locality inside the regions. Relative to the average UCC, all these sediments are depleted in major and trace elements; only SiO_2_ is enriched, showing that these are matured siliciclastic sediments of different grades. The greatest depletion is observed in the NWHR in the Babót samples, where all major- and trace-element concentrations are much lower than in other mines. It is conspicuous that the Babót samples are much depleted in Ca and enriched in Mg compared to other samples of the NWHR, reflecting a higher amount of dolomite in the carbonatic components of the sedimentary beds [12]. While in the SWHR the Ca and Mg concentrations are more variable, the Mg concentration is still higher, reflecting the presence of dolomite. At the MDR, the situation is the opposite: the Ca concentration is higher relative to Mg, indicating the dominance of limestone. At the NEHR there is very little Ca, and the Mg concentration is under the detection limit, reflecting the fact that there are no carbonatic rocks in the sediments. Enrichment of some major (especially Ti, Al, Na, K) and trace elements in the sand (0–2 mm), and very fine (2–4 mm) and fine gravels (4–8 mm) is due to the clay content. This phenomenon is the most prominent in the SWHR.

Elements in the gravel and sand samples that form long-lived isotopes after neutron capture (Ce, Hf, Fe, Sb, Tb, Sc, Ta, Cs, Co, and Eu) are below the elemental concentrations typical of the average Portland cement and also depleted compared to the upper continental crust (UCC). Looking at the four different mining regions, the NEHR and the MDR show similar element distribution patterns. The greatest variety, but still the most depleted samples, are from the NWHR, and, particularly, in Babót. The well-developed sedimentary rocks with high SiO_2_-content and a lower amount of neutron-induced long-lived isotopes are without particular risk, and thus they are fully compliant to be used in shielding concrete. 

The very-long-lived isotopes are also below 10 µg/g in all examined samples, and thus they do not significantly contribute to the activation. As the modelling also revealed, the composition of the investigated sand and gravels in Hungary does not show outliers that would significantly affect activation properties. Even elements with long-lived activity in the sand and gravel samples will decay at the latest 30 years after the end of irradiation.

## 6. Conclusions

All ingredients utilized in radiation shielding concrete must ultimately lead to the minimization of radioactivation. Sand and gravels, as the major constituents of concrete, were systematically analysed for elemental composition, from four major gravel-mine regions (NWHR, SWHR, MDR, NEHR) of Hungary, considering different grain-size fractions (0–4; 4–8; 8–16; 16–32 mm). Results reflected a depleted composition of mature silica-rich sediments. Based on these elemental concentration fingerprints, the claimed origin of an industrial raw material can be validated on a regional scale. The concentrations of the long-lived isotope-forming elements were compared to the average trace-element content of the examined types of Portland cement. The concentration of the long-lived isotopes is higher in Portland cement than in the sand and gravel. The outcome of the geochemical survey pointed towards the conclusion that, in general, there are small amounts of trace elements with long half-lives (2–13.5 y) in the examined aggregate constituents of concrete. Most sand and gravel quarried in Hungary is potentially suitable for preparing nuclear-grade shielding concrete; however, petrological, and geochemical studies revealed that the gravel and sand from the NW Hungarian region have the lowest amount of activation precursors. From methodological aspects, this database has highlighted the complementarities and the synergies between the two nuclear analytical methods. The basic principles of the nuclear analytical methods just reflect the real situation inside the radiation shielding materials close to the reactor. Thus, it is advantageous to use these techniques for the elemental analysis of radiation susceptibility studies. 

The elemental concentration data were used as input for the FISPACT code to model changes in the activity of the samples in time. The radiation field typical of the near-vessel area of a nuclear research reactor was assumed for 10 years of use, equivalent to 50 years of service in a power plant. Subsequently, we followed the dose rate of major contributor isotopes for up to 100 years following the reactor shutdown. The aggregates, sand, and gravels contribute only to a small extent to the total activity, and they decay to the clearance level in 30 years, which is a reasonable time interval for decommissioning [49].

The selection of adequate materials is largely based on the concentrations of the long-lived isotope precursors they bring into the concrete. FISPACT modelling calculates the whole decay scheme of the activated elements and allows predictions of activities for any time instance in the future to be made. 

## Data Availability

Not applicable.

## Supplementary Materials

The following supporting information can be downloaded at: https://www.mdpi.com/article/10.3390/ma16093520/s1, Figure S1: The PGAA spectrum was collected in a 12 MeV range, containing 700–1000 peaks. On the X-axis the energy (keV) and on the Y-axis the intensity (in log scale) is shown; Figure S2. The NAA spectrum has been measured in a 3 MeV range, containing 100–150 peaks. This spectrum has been collected shortly after irradiation (4 days). On the X-axis the energy (keV) on the Y-axis the intensity (in log scale) is shown; Figure S3. The NAA spectrum has been measured at a longer decay time (3 weeks after the irradiation) to improve the detection limits for several medium- and long-lived radionuclides. After the dominant short-lived isotopes had decayed, the long-lived radionuclides could be better determined.
