# Supplementary material for: Hungarian Fine-to-Coarse Aggregate, a Possible Constituent of Near-Vessel Structural Concrete of Nuclear Power Plants
Source: Materials (Basel). 2023 May 4;16(9):3520. doi: 10.3390/ma16093520 (PMC10179888; doi:10.3390/ma16093520)
Supplement: Supplementary file 1 [file materials-16-03520-s001.zip › materials-2300602-supplementary.pdf]

We present typical NAA and PGAA spectra connected to the article:

Katalin Gméling, Veronika Szilágyi, Ildikó Harsányi, and László Szentmiklósi: Hungarian fine-to-coarse aggregate, a possible constituent of near-vessel structural concrete of nuclear power plants

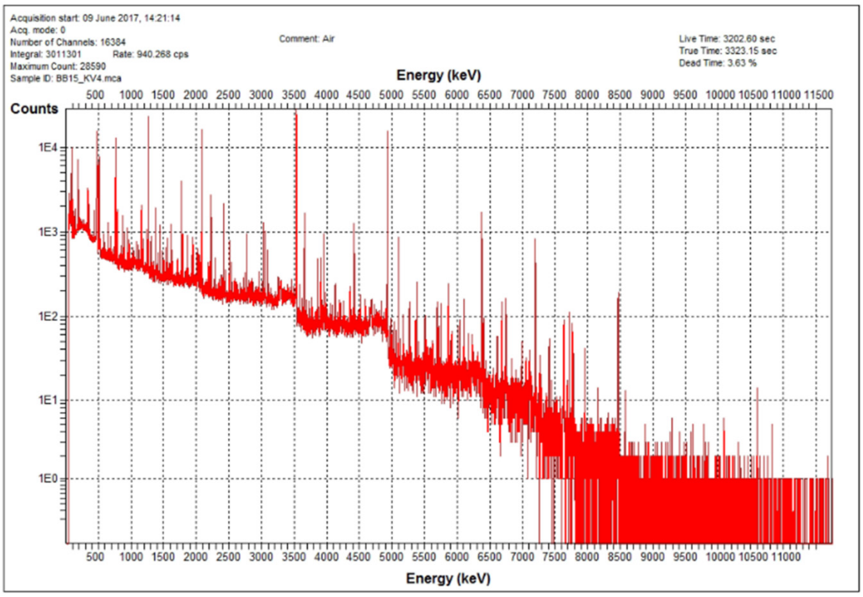

Figure S1. The PGAA spectrum was collected in a 12 MeV range, containing 700-1000 peaks. On the X-axis the energy (keV), and on the Y-axis the intensity (in log scale) is shown.

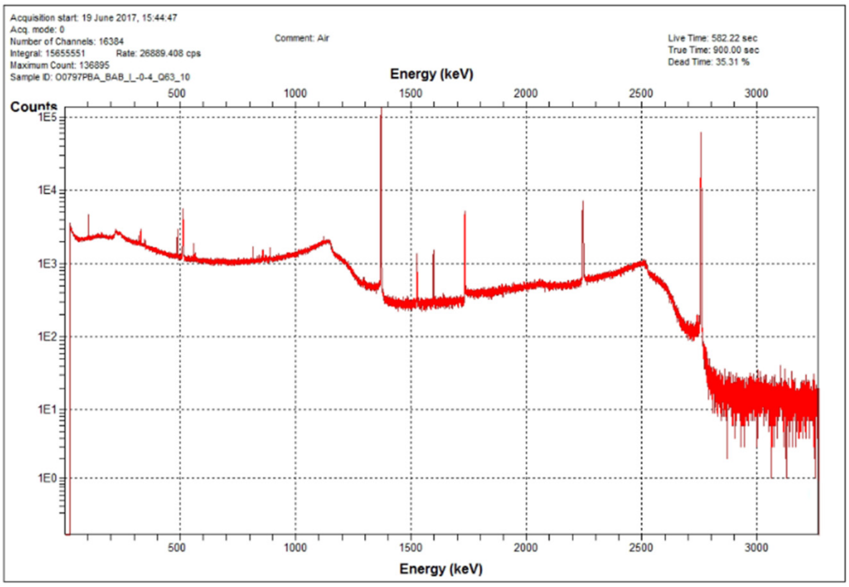

Figure S2. The NAA spectrum has been measured in a 3 MeV range, containing 100-150 peaks. This spectrum has been collected shortly after irradiation (4 days). On the X-axis the energy (keV) on the Y-axis the intensity (in log scale) is shown.

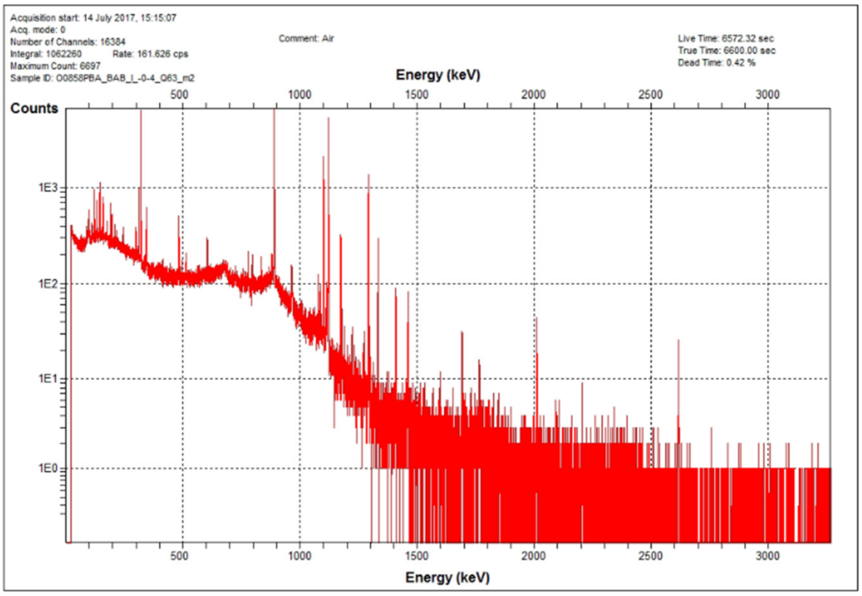

Figure S3. The NAA spectrum has been measured at a longer decay time (3 weeks after the irradiation) to improve the detection limits for several medium- and long-lived radionuclides. After the dominant short-lived isotopes had decayed, the long-lived radionuclides could be better determined.
